# Computational Modeling on Drugs Effects for Left Ventricle in Cardiomyopathy Disease

**DOI:** 10.3390/pharmaceutics15030793

**Published:** 2023-02-28

**Authors:** Smiljana Tomasevic, Miljan Milosevic, Bogdan Milicevic, Vladimir Simic, Momcilo Prodanovic, Srboljub M. Mijailovich, Nenad Filipovic

**Affiliations:** 1 Faculty of Engineering, University of Kragujevac, 34000 Kragujevac, Serbia; 2 BioIRC Bioengineering Research and Development Center, 34000 Kragujevac, Serbia; 3 Institute for Information Technologies, University of Kragujevac, 34000 Kragujevac, Serbia; 4 FilamenTech, Inc., Newton, MA 02458, USA; 5 BioCAT, Department of Biology, Illinois Institute of Technology, Chicago, IL 60616, USA

**Keywords:** cardiomyopathy heart modelling, hypertrophic cardiomyopathy (HCM) patients, dilated cardiomyopathy (DCM) patients, fluid–structure interaction (FSI), kinetic processes of sarcomeric proteins interactions, disopyramide, digoxin, mavacamten, 2-deoxy adenosine triphosphate (dATP), modeling drug influence

## Abstract

Cardiomyopathy is associated with structural and functional abnormalities of the ventricular myocardium and can be classified in two major groups: hypertrophic (HCM) and dilated (DCM) cardiomyopathy. Computational modeling and drug design approaches can speed up the drug discovery and significantly reduce expenses aiming to improve the treatment of cardiomyopathy. In the SILICOFCM project, a multiscale platform is developed using coupled macro- and microsimulation through finite element (FE) modeling of fluid–structure interactions (FSI) and molecular drug interactions with the cardiac cells. FSI was used for modeling the left ventricle (LV) with a nonlinear material model of the heart wall. Simulations of the drugs’ influence on the electro-mechanics LV coupling were separated in two scenarios, defined by the principal action of specific drugs. We examined the effects of Disopyramide and Dygoxin which modulate Ca^2+^ transients (first scenario), and Mavacamten and 2-deoxy adenosine triphosphate (dATP) which affect changes of kinetic parameters (second scenario). Changes of pressures, displacements, and velocity distributions, as well as pressure–volume (P-V) loops in the LV models of HCM and DCM patients were presented. Additionally, the results obtained from the SILICOFCM Risk Stratification Tool and PAK software for high-risk HCM patients closely followed the clinical observations. This approach can give much more information on risk prediction of cardiac disease to specific patients and better insight into estimated effects of drug therapy, leading to improved patient monitoring and treatment.

## 1. Introduction

Cardiomyopathies are defined as structural and functional abnormalities of the ventricular myocardium [1]. When the same genetic mutation happens in more than one family member, it is called familial cardiomyopathy (FCM). On the other hand, in the absence of relevant family history, nonfamilial cardiomyopathy is classified [2]. There are four major classifications of cardiomyopathy: hypertrophic (HCM), dilated (DCM), restrictive (RCM), and arrhythmogenic right ventricle (RV) cardiomyopathy (ARVC) [3], while the most frequent are HCM and DCM.

HCM can cause outflow obstruction and abnormal movement of the mitral valve due to increased left ventricle (LV) wall thickness, which is called left ventricular outflow tract obstruction (LVOTO). It can be observed that approximately 70% of patients with HCM have LVOTO with severe basal septal hypertrophy and systolic anterior motion of the mitral valve [4].

Understanding of cardiac muscle activity in HCM and DCM cardiomyopathies has been significantly improved with continuous development and integration of computational models [5]. Furthermore, the idea of an integrative modeling methodology by coupling several spatial and temporal scale computational tools presented in the study of Prodanovic et al. [6] might help in identifying the symptoms and outcomes of patients with multiple genetic disorders. For the simulation of total heart health or pathology, molecular, cellular, tissue, and organ levels have to be integrated. However, simulations of muscle function at the organ level [7] with even simpler models [8] require a lot of high-performance computing and memory [9]. Furthermore, simulation of fluid–structure interactions (FSI) in whole heart during the total heart cycle demands usage of a large number of finite elements (FEs) due to complex heart geometry. Moreover, calculating precise muscle characteristics for specific patients’ conditions, such as muscle stiffness and active tension, requires solving partial differential equation solutions [10,11] or using Monte Carlo approaches [10,11,12]. Nevertheless, high-performance computing makes it possible to run multiscale models of the heart, in which the behavior of each cell is controlled by molecular mechanics and organ level tissue structures using the FE method. In this way, heart simulations might also be used for clinical applications besides the scientific purposes. In terms of clinical application, Siguira et al. [13] used UT-Heart models for cardiac resynchronization therapy and surgery for congenital heart disease, while the computational platform for in silico clinical trials (SILICOFCM platform [14]) has been used for risk prediction of cardiac hypertrophic disease [15,16]. The SILICOFCM platform integrates patient-specific data and allows the testing and optimization of medical treatment to maximize positive therapeutic outcomes. The integrated data include biological, genetic, and clinical imaging data which are processed using various approaches such as bioinformatics, machine learning, data analytics, multiscale modelling, and FE modelling.

The FE method can be applied standalone or coupled with different computational methods for modelling complex biological tissue behavior and treatments. In a recent study, the FE method [17] was successfully applied for the simulation of liver tumor treatment also combining magnetic field and heat control. Treatment of liver cancer by destroying the damaged liver tissue using a designed surgical needle was also modelled under a local heating process [18]. The utilized Runge–Kutta and finite difference method were integrated in a hybrid model for numerical analysis of hyperthermia treatment of tumor or cancer cells [19], as well as for numerical analysis of thermal response of a multi-layer skin model under heating and cooling processes [20]. The advantage of these computational methods is their wide application in tissue modelling, from the micro to macro level.

On the micro level of cardiac tissue, micromechanics models based on the regulatory and contractile proteins in the sarcomeres analyzed the generation of force in the cardiac muscle tissue. They include the steady-state force–calcium, force–length, and force–velocity relationships and the length-dependent prolongation of twitches and increase in peak force [21].

This study, as part of the SILICOFCM in silico clinical trial, brings novelty in multiscale examination of drug interactions applying coupled macro- and micro-simulation through FE modeling of FSI and molecular drug interactions with cardiac cells. With this approach, adverse drug effects can be avoided, sudden cardiac death can be prevented, and the time needed for the desired result of drug treatment can be shortened. The main motivation for such an approach relies on improvement of computational modeling in testing the effects of pharmacological treatment, aiming to reduce animal experiments and human clinical trials. Moreover, computer-aided drug design is a well-known approach in the production strategy of drugs [22]. It can significantly reduce time and costs for procedures in drug research and development [23]. Additionally, pharmacophore modeling, virtual screening, molecular docking, and molecular dynamic simulations are becoming very popular in silico techniques [24].

In this study, an FSI algorithm for FE simulation was firstly introduced, and then an algorithm of the FE model was integrated with stretches along muscle fibers. The principal actions of specific drugs were divided into two major groups. The first class of drugs modulates calcium transients (Disopyramide and Digoxin), while the second class changes the kinetics of contractile proteins (Mavacamten, 2-deoxy adenosine triphosphate (dATP)). We quantitatively assessed the effects of drugs Disopyramide, Digoxin, Mavacamten, and dATP on the pressure and volume changes in the LV models for relevant HCM or DCM patients. Results for pressure, displacement, and velocity distribution, as well as P-V loops for LV HCM and LV DCM patients for basic condition (without administered drugs) and with effects of using respective drugs are presented. Additionally, results from the Risk Stratification Tool and PAK software for high-risk HCM patients are presented and compared with available clinical observations.

## 2. Materials and Methods

### 2.1. Fluid–Solid Coupling

The movement of fluid in the left ventricle can be considered as a laminar flow of the incompressible fluid, which is described using the continuity equation and Navier–Stokes equations:

(1) $$-\mu \nabla^{2}v_{l}+\rho \left(v_{l}\cdot \nabla \right)v_{l}+\nabla p_{l}=0,$$

(2) $$\nabla v_{l}=0,$$

where $v_{l}$ is the blood flow velocity, $p_{l}$ is the pressure, μ is the coefficient of dynamic viscosity of blood, and ρ is the density of blood. These equations can be transformed into the balance equations of an FE by using the Galerkin method. The incremental-iterative balance equation of an FE for a time step ‘*n*’ and equilibrium iteration ‘*i*’ has the form

(3) [1ΔtM+K˜vv(i−1)n+1KvpKvpT0]{ΔV(i)ΔP(i)}blood={Fext(i−1)n+10}−[1ΔtM+K(i−1)n+1KvpKvpT0]{V(i−1)n+1P(i−1)n+1}+{1ΔtMVn0}

where V(i−1)n+1 and P(i−1)n+1 are the nodal vectors of blood velocity and pressure, with the increments in time step $\Delta V^{\left(i\right)}$ and $\Delta P^{\left(i\right)}$; Δt is the time step size and the left upper indices ‘*n*’ and ‘*n* + 1’ denote the start and end of the time step.

Using velocities as nodal variables, the incremental-iterative equations of the force-balance for a FE and per unit volume can be written in the usual form:

(4) $$\left(\frac{1}{\Delta t}M+{\bar{K}}^{\left(i-1\right)}\right)\Delta V^{\left(i\right)}=F^{ext(i)}-F^{\mathrm{int}(i-1)}-\frac{1}{\Delta t}M\left(V^{\left(i-1\right)}-V^{t}\right),$$

where Δt is the time step, *i* is the iteration counter, and $F^{ext(i)}$ are external nodal forces acting on the element; $V^{\left(i-1\right)}$ and $V^{t}$ are nodal velocities at a previous iteration and at the start of the time step, respectively. The mass and stiffness matrices are

(5) $$M=\rho N^{T}N,{\bar{K}}^{\left(i-1\right)}={\left({\bar{B}}^{T}C_{T}\bar{B}\right)}^{\left(i-1\right)},$$

where ρ is the mass density, and the vector of the internal nodal forces is

(6) $$F^{\mathrm{int}(i-1)}={\bar{B}}^{T(i-1)}{\bar{\sigma }}^{\left(i-1\right)}.$$

The tangent constitutive matrix, $C_{T}^{\left(i-1\right)}$, in the local system will be determined within the computational procedure presented below.

There are two approaches for the FE modeling of FSI problems: (a) strong coupling method, and (b) loose coupling method. For the strong coupling, the solid and fluid domains are modeled as one mechanical system. In the loose coupling, solid and fluid domains are modeled separately with different FE solvers. Namely, the solid domain is modelled in a computational solid dynamics (CSD) solver and the fluid domain in a computational fluid dynamics (CFD) solver. Although the solutions are obtained with different FE solvers, the parameters from one solution which affect the solution for the other medium are transferred successively.

There is no slip between the fluid and solid at the common boundary, which means that the nodes at the solid–fluid boundary have the same displacements and velocities for the solid and fluid domains. If the strong coupling approach is used, the terms of the FE matrices and forces corresponding to these common nodes are summed as is usual in the FE assembling procedure. In the loose coupling method, the systems of balance equations for the two domains are formed separately and there are no such computational difficulties. Both strong and loose coupling are available in our PAK FE software package [25]. Similar results can be achieved with both methods but since the loose coupling is computationally less intensive, this method is used more frequently. In loose coupling, the equations are first solved for the fluid domain. When the convergence for the fluid domain is reached, the nodal forces, for an element *E*, which has nodes at the boundary, is calculated as in (6):

(7) $$F^{E}=\left[\begin{array}{cc} \frac{1}{\Delta t}M+K_{vv} & K_{vp}^{} \end{array}\right]\left\{\begin{array}{c} V\\ P \end{array}\right\},$$

and the forces at the common boundary as the vector $F^{E}$ are used.

### 2.2. Finite Element (FE) Solvers

**Figure 1:**
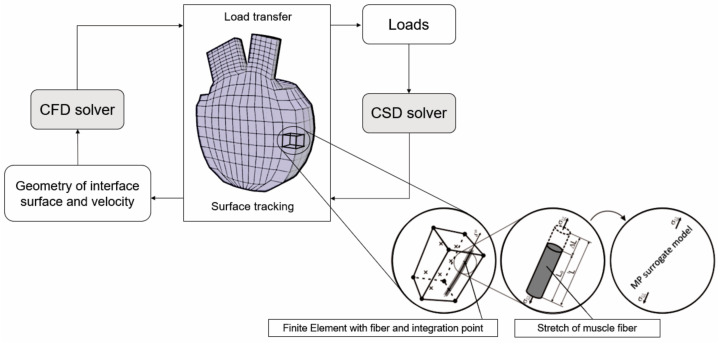
Block diagram of the FSI algorithm coupled with multi-scale model of muscle contraction. Muscle is discretized into FEs where each FE contains muscle fiber and integration points [26].

This section explains how FE model was integrated with stretches along muscle fibers. A multi-scale model of muscle contraction together with graphical interpretation of the algorithm for the FSI problem is shown in Figure 1 [26].

**Figure 2:**
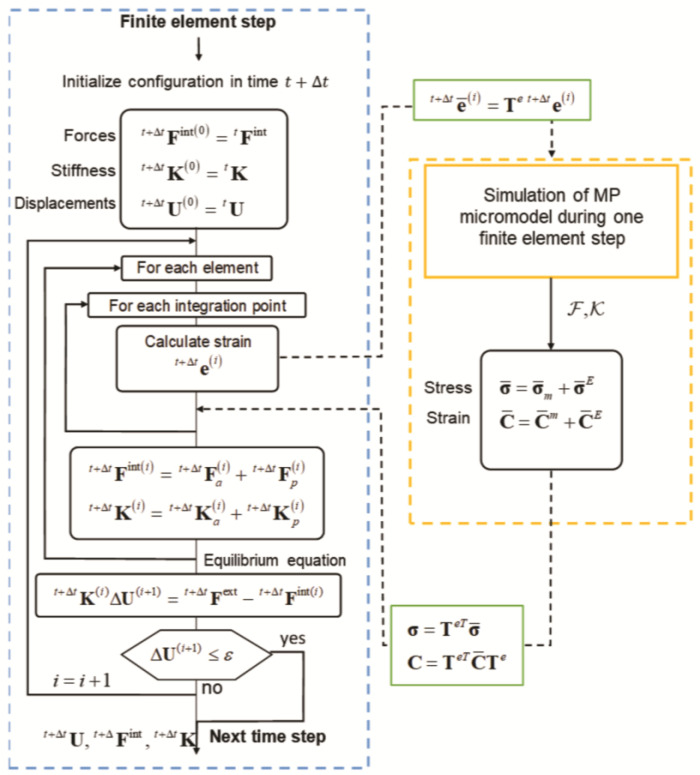
Algorithm linking FE analysis and MP surrogate model.

Based on input parameters, the current state of the material of the microscale model, and provided stretch, in each iteration *i*, the Mijailovich–Prodanovic (MP) surrogate model of sarcomere contractions [6,16] calculates the local active tension and instantaneous stiffness along muscle fibers (Figure 2). Afterwards, the macroscale model solves the equilibrium equation that includes local active tension and stiffness from the MP model and provides the stretch and total tension in the FE integration points. This coupled incremental iterative process is repeated until the changes in the velocity and the active tension at the current iteration are below the prescribed tolerances.

### 2.3. Cardiomyopathy Risk Stratification

This section briefly explains the Cardiomyopathy Risk Stratification Tool, developed as part of the SILICOFCM platform, which uses specialized data mining methods for supervised learning to provide an identification of high-risk patients [14,15,16,27]. More specifically, its main aim is to identify patients with a high risk of severe events such as sudden cardiac death (SCD) or life-threatening arrhythmias. In addition to modeling patient risk, the tool is also supplemented with reliability estimates for risk predictions. Both the risk prediction model and reliability estimates allow medical experts to decide whether the patient will be subjected to further analysis, and how trusting the automatically predicted risk level is.

The developed Risk Stratification Tool uses machine learning methods for supervised and unsupervised learning to mine heterogeneous patient data provided by clinical partners within the SILICOFCM clinical study. The final model stratifies patients into a low-risk or high-risk class based on the probability that one or more of the selected severe events (e.g., SCD, heart failure, life-threatening arrhythmias) will occur in the next five years. The results of the Risk Stratification Tool for five HCM patients (selected in a such way that higher and lower risk are present in the population) are presented in Section 3.1.

### 2.4. Drug Modeling

Despite the lack of understanding of the disease’s progression, there is significant evidence that mutations frequently cause the disease. There have been numerous in vitro investigations of the characteristics of mutated proteins, as well as studies of muscle from transgenic mice with these mutations. However, there is now a big gap between understanding how the mutation modifies the behavior of the protein and how this process leads to disease. Part of the complication stems from the fact that the protein is present in the tissue from birth, although there are often no observable changes in phenotype until adolescence or even later.

Until recently, there had been no method to connect the in vitro studies of individual proteins carrying mutations with studies of intact systems—either trans-genetic mice or data from human-tissue- or patient-specific data. However, recently developed computational models, such as the MUSICO platform [28], have significantly advanced our understanding of cardiac muscle activity in HCM and DCM cardiomyopathies [5]. The MUSICO platform can trace the effects caused by genetic mutations from the molecular level and across multiple length and time scales up to the muscle fibers and translate these effects from rodent studies to human muscle behavior [29]. This is a powerful tool which can also be used to assess the effects of small molecules (drugs) on muscle contraction and determine specific pathways of drug action.

Using MUSICO simulations, we have recognized two major groups by the principal action of specific drugs. The first group of the drugs modulates calcium transients, while the second group of the drugs changes kinetics of contractile proteins. Each group can be further divided into two different subgroups depending on cardiomyopathy type (HCM or DCM). Disopyramide is, for example, used for HCM modulation of [Ca^2+^] transients because it lowers peak and baseline levels of [Ca^2+^] [30]. On other hand, for DCM, Digoxin increases the peak of [Ca^2+^] transient during twitch contractions, but without influencing time to peak and relaxation times [31].

Furthermore, drugs such as Mavacamten and 2-deoxy adenosine triphosphate (dATP) change the kinetics of contractile proteins. Mavacamten has been used for treating HCM because it is associated with the regulation of transition rates between ordered parked (OFF) states and disordered myosin detached (ON) states [32]. On the other hand, dATP is a promising drug for treating DCM which modulates crossbridge cycle rates and affects structural OFF/ON transitions of myosin heads [33,34,35].

#### 2.4.1. Drugs That Modulate [Ca^2+^] Transients

**Figure 3:**
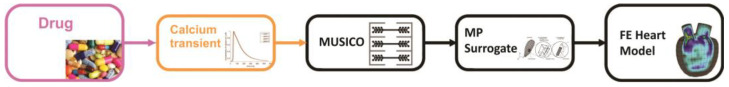
Scenario 1: Drug action via modulation of calcium transient. Adopted from ref. [16].

An example of a scenario of in silico testing of drugs that modulate calcium transients has been presented in Figure 3. In this scenario, the drug is acting through changes in ionic currents or membrane (channels) properties. This, in turn, modulates intracellular calcium concentration during muscle contraction. To model these effects, experimentally observed calcium transients were used as the inputs for MUSICO and MP surrogate models where available.

- Disopyramide

On a macro level, disopyramide reduces LVOT gradients, with a slight decrease in the resting ejection fraction. It has been observed that Disopyramide decreases early drag forces on the mitral valve. Additionally, Disopyramide results in a modest reduction of global systolic function—5% to 6%. 

**Figure 4:**
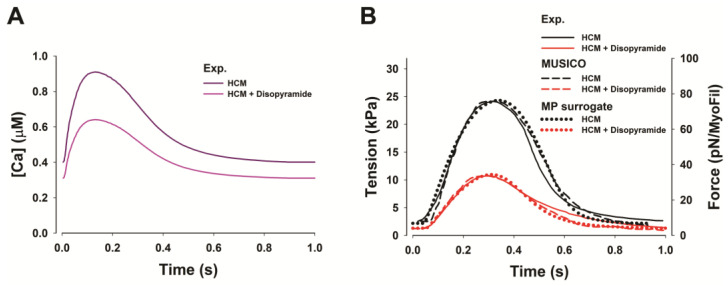
(**A**) Adjusted calcium transients from the observed in human HCM trabeculae [30]. (**B**) Disopyramide effects on human HCM trabeculae twitch contraction, shown as comparison between the experimental twitch tension traces [30] (*solid lines*), and the predictions by MUSICO (*dashed lines*) and MP surrogate model (*dotted lines*) in presence of (*red*) and without Disopyramide (*black*). Figure is adopted from Prodanovic et al. [6].

Experimental observations of normal and HCM human cardiac tissues treated with disopyramide [30] showed that the drug decreases intracellular calcium transient by both decreasing the twitch [Ca^2+^] peak and the level of basal calcium concentration (Figure 4A). In the recent study by Prodanovic et al. [6], it had been demonstrated that MUSICO simulations of human trabeculae twitches can predict a decrease in the peak twitch tension by ~55% and a decrease in the resting tension by ~50% in the presence of 5 µmol/L of Disopyramide (Figure 4B), matching the observations of Coppini et al. [30].

b. Digoxin

Cardioactive glycosides (e.g., Digoxin) have been important in treating congestive heart failure for more than 200 years, in large part because of a positive inotropic effect. The effect of Digoxin results in higher diastolic [Ca^2+^], higher SR Ca^2+^ content, and even greater Ca^2+^ influx via NCX during the action potential which increases twitch tension [36].

**Figure 5:**
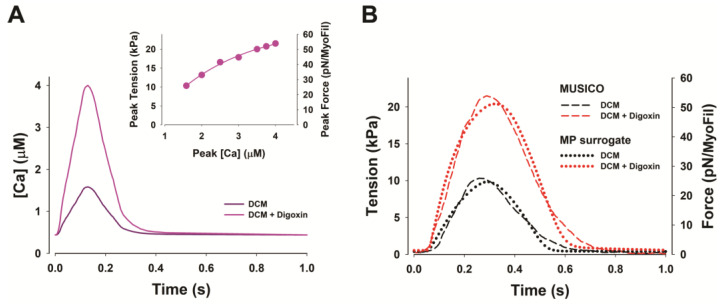
Digoxin effects on human DCM trabeculae twitch contraction. (**A**) Calcium transient for DCM in presence of (*pink*) and without Digoxin (*purple*). Inset in (**A**) peak tension dependence on peak calcium concentration during twitch contractions in presence of increased doses of Digoxin. (**B**) MUSICO (*dashed lines*) and MP surrogate model predictions (*dotted lines*) in presence of (*red*) and without Digoxin (*black*).

It is observed that DCM causes an enlargement of the chambers while the muscular wall progressively becomes thinner. In addition, cardiac function in DCM is compromised with decreased cardiac muscle contractility that, along with the structural changes of an enlarged left ventricle, reduces systolic function, with an ejection fraction <50%. Digoxin increases the intracellular calcium concentration transient (opposite of Disopyramide) by increasing the [Ca^2+^] peak during twitch contractions but keeping the time to peak and the relaxation time unchanged [31,37] as illustrated in Figure 5A.

The increase in intracellular calcium concentration increases heart wall tension and, therefore, increases systolic pressure and ejection fraction. The MUSICO simulations of human DCM trabeculae twitches predicted a dose-dependent increase in the peak twitch tension up to twofold (Inset in Figure 5A), similar to the observations of Morgan [36]. Furthermore, MUSICO and MP surrogate model predictions of twitch tension transients for human DCM fibers in presence of and without Digoxin are presented in Figure 5B.

The majority of parameters used in MUSICO simulations originate from wild-type (WT) human trabeculae with 100% β myosins used in the study of Prodanovic et al. [29], while calcium transients are taken from mouse DCM [5] and adapted to human. The FE simulations of human LV using PAK solver [25] enable quantitative assessment of the effect of Digoxin on cardiac output including increase in both systolic and diastolic pressures, and the ejection fraction.

#### 2.4.2. Drugs That Affect Changes in Kinetic Parameters

**Figure 6:**
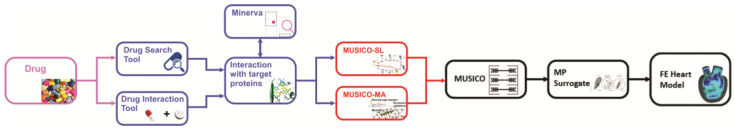
Scenario 2: Changes in kinetic of contractile proteins for drug action. Adopted from ref. [16].

The second scenario for testing drugs that affect changes in the kinetic characteristics of protein interactions is shown in Figure 6.

- Mavacamten

Mavacamten (MYK-461) is an allosteric inhibitor of cardiac myosin ATPase which reduces actin-myosin cross-bridge formation. In experimental HCM models it has been shown that this drug directly reduces myocardial contractility and improves myocardial energetic consumption in experimental HCM models. A clinical randomized trial demonstrated the efficacy and safety of Mavacamten in reducing left ventricular outflow tract obstruction and ameliorating exercise capacity [38]. Mavacamten reduces adenosine triphosphatase activity in cardiac myosin heavy chain. This has a consequence in reduction of the contraction of the heart, which can contribute to improving obstruction in HCM patients [39].

Mavacamten has already been successfully used in clinical trials for treatment of HCM and adopted by the U.S. Food and Drug Administration (FDA) [40]. Mavacamten’s negative inotropic action is likely mediated by the shift of detached motor heads towards an autoinhibited SRX state. The impact of Mavacamten on cardiomyocyte electrophysiology and Ca^2+^ handling is still under investigation, but the drug is able to reverse the adverse remodeling of cardiomyocyte excitation–contraction coupling observed in mouse models of HCM [39]. 

The effect of increased tension in HCM can be attenuated by Mavacamten’s action on the level of crossbridge cycle, specifically by modulation of the state transition rates between the SRX state and the disordered myosin detached states, capable of binding to actin. The recent experimental observations of Ma et al. [32] have shown that there is a significant decrease in tension (~33%) in steady-state force development in skinned porcine heart muscles in the presence of 1 μM Mavacamten.

The effects of Mavacamten on human trabeculae were preliminary assessed with MUSICO simulations by using the same calcium transient as observed in HCM. The simulations predicted a similar decrease in tension, ~30% for steady-state force development at high [Ca^2+^] and about 50% in twitch contractions. Furthermore, simulations also showed a significant decrease in resting tension, which is the expected outcome of Mavacamten treatment. These changes are similar to the tension responses predicted by Disopyramide, but the mechanisms of action of these two distinctive drugs are fundamentally different.

It has been observed that Mavacamten is related to a nearly complete resolution of mitral valve systolic anterior motion. This can be directly associated with a reduction in the LVOT gradient. Additionally, a decrease in left ventricle mass index, left atrial volume index, and lateral E/e’ have also been associated with the influence of Mavacamten. 

On the other hand, Mavacamten has not been associated with cardiac structural changes such as reductions in interventricular septum thickness or left ventricle end-diastolic diameter. However, there are observations for significant changes in inferolateral wall thickness and left ventricle end-systolic diameter [41].

These data are suitable for FE analysis, i.e., for simulations of the heart using PAK solver [25], to quantitatively assess the effect of Mavacamten on cardiac output including decrease in both systolic and diastolic pressures and the ejection fraction.

b. 2-Deoxy Adenosine Triphosphate (dATP)

The molecule dATP can replace ATP as the energy source for the motor protein myosin contraction in striated muscle. dATP allosterically enhances myosin crossbridge binding to actin (and cycling kinetic) such that small amounts of dATP are potent [42,43]. Increasing the cardiomyocyte level of dATP from the typical <0.1% of the ATP pool to just 1% is enough to significantly increase contraction [44]. Through either viral vector or transgenic approaches this results in increased dATP levels sufficient to increase contractile magnitude and kinetics. Thus, approaches to increasing cardiomyocyte dATP constitute an exciting and novel therapy with the potential to treat heart failure.

The significant increase in cardiac muscle contractility could be beneficial for DCM cardiomyopathies. The effects of dATP on DCM mice trabeculae were preliminary assessed with MUSICO simulations at the level of muscle fiber [6].

## 3. Results

### 3.1. Cardiomyopathy Risk Stratification Tool

**Figure 7:**
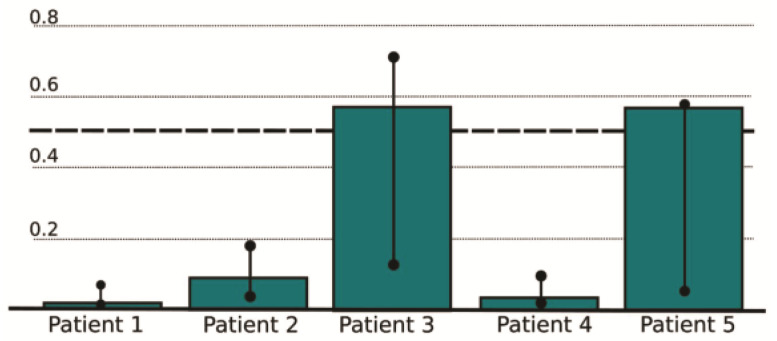
Risk prediction report from the Risk Stratification Tool for the selected five patients.

Five HCM patients who have not been used for training the machine learning model were randomly selected but in a such way that higher and lower risk are present in the population. The testing which was performed on the five selected patients provided the following results: two patients were at high risk (patients No. 3 and 5), whereas three patients were at low risk (patients No. 1, 2, and 4). The detailed reports from the Risk Stratification Tool are displayed in Table 1 and Figure 7. The prediction bars for the five selected patients are included in Figure 7. Insight into real-life data from clinical records and follow-up of patients No. 3 and 5 who were at high risk according to the results from the Risk Stratification Tool showed that their real health condition was worse, especially in case of patient 3 (heart failure symptoms).

**Figure 8:**
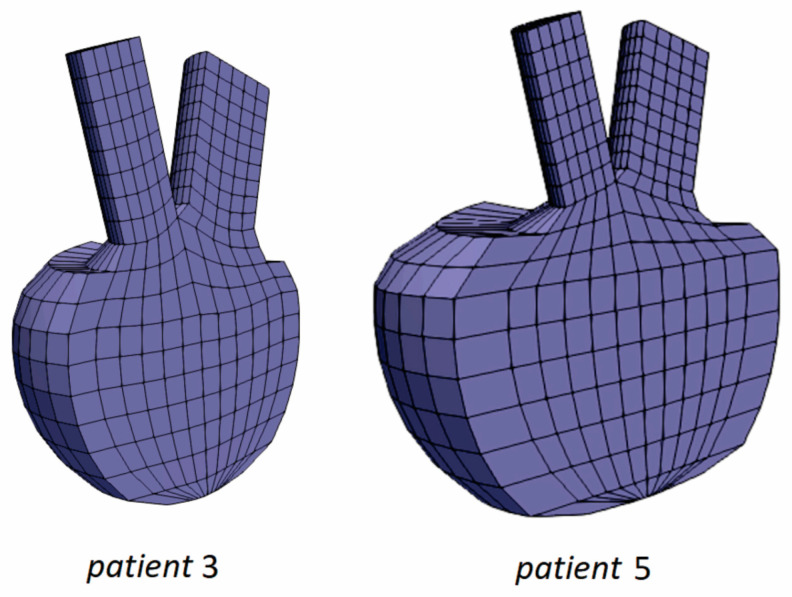
Left ventricle geometries for patient 3 and patient 5.

**Figure 9:**
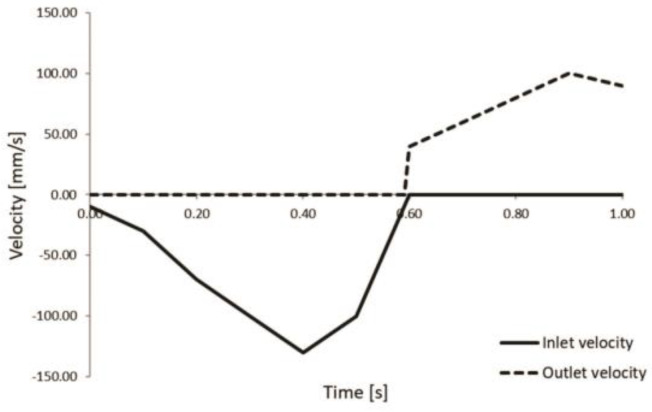
Prescribed inlet and outlet velocities for aortic output and mitral input.

In addition to the Risk Stratification Tool, we employed PAK FE simulation for patients 3 and 5, who were at high risk of severe events. The LV geometries of the patients 3 and 5 are shown in Figure 8, respectively. Compared to patient 3, patient 5 has a larger LV with thicker walls. Prescribed inlet and outlet velocities for aortic output and mitral input are presented in Figure 9.

It should be emphasized that our parametric geometrical models are simplified but reflect the features of patient-specific measurements, i.e., the geometrical parameters were obtained from the patient data [16]. Measured valve diameters and wall thicknesses were used to generate the FE mesh of our parametric LV model. The lengths of the mitral and aortic branches do not influence the results of the computation. We adopted nominal inlet and outlet velocity values (shown in Figure 9) and scaled them according to the size of the valve diameters. Inlet velocity is scaled proportionally to the mitral valve diameter, and outlet velocity is scaled proportionally to the aortic valve diameter.

**Figure 10:**
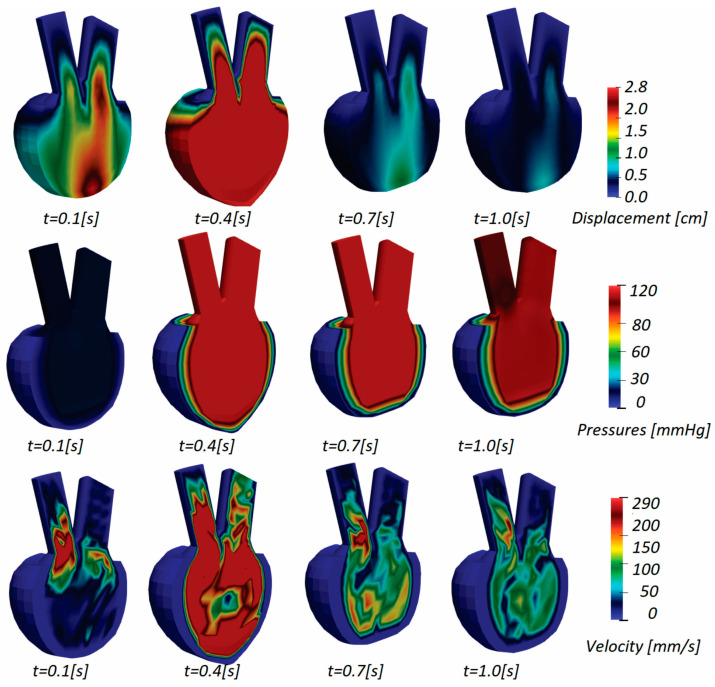
Displacement, pressures, and velocity fields for patient 3.

The results obtained for patient 3 are shown in Figure 10, presenting displacements, pressures, and velocity distribution. Since the injection part of the cycle occurs during the initial couple of steps, displacements at the mitral valve and base part of the model are noticeable. When the contraction occurs and the fluid begins to flow out (t = 0.7 s), the bottom half of the wall experiences the most deformation. The solid wall gradually returns to its original state (t = 1.0 s) and deformations decrease over the remaining time.

During diastole, the fluid is injected into the ventricle and its volume increases, and pressure is maximum at the mitral valve (t = 0.4 s) throughout the first part of the cycle. When the injection cycle is finished and the mitral valve is closed, the ventricle contracts and ejects fluid through the aortic valve, resulting in the highest pressure value in the model until the end of the cycle.

**Figure 11:**
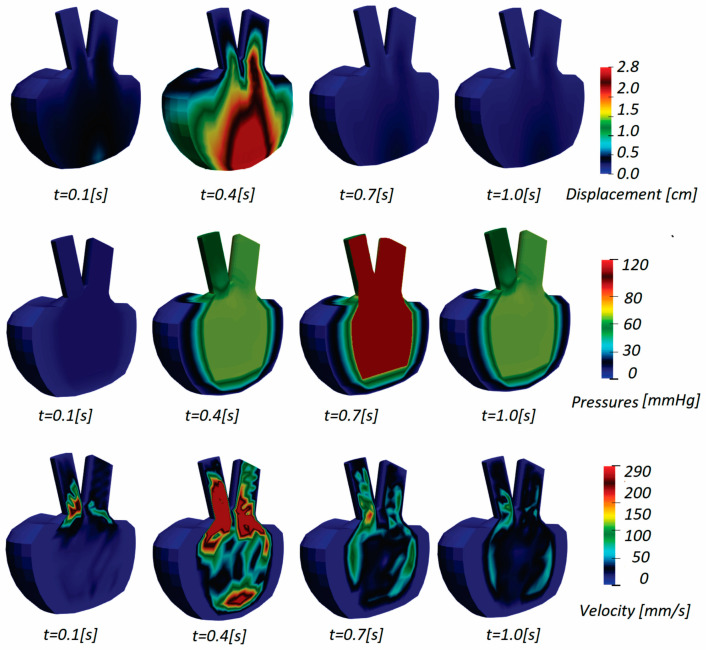
Displacement, pressures, and velocity fields for patient 5.

Displacements, pressures, and velocity fields for patient 5 are presented in Figure 11. The displacements are the largest at the middle of the diastole during the blood pumping. Towards the end of the cardiac cycle, the LV model returns to the initial configuration.

**Figure 12:**
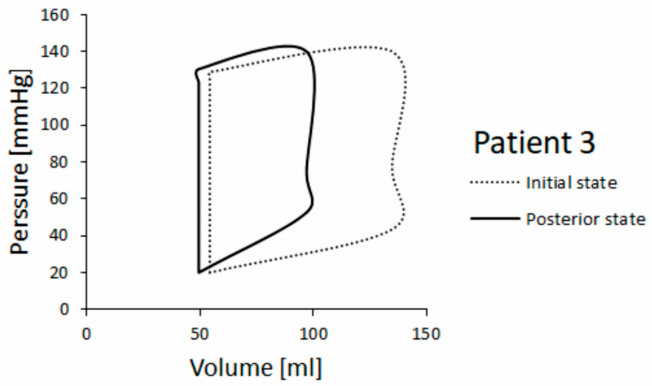
P-V diagram for patient 3.

**Figure 13:**
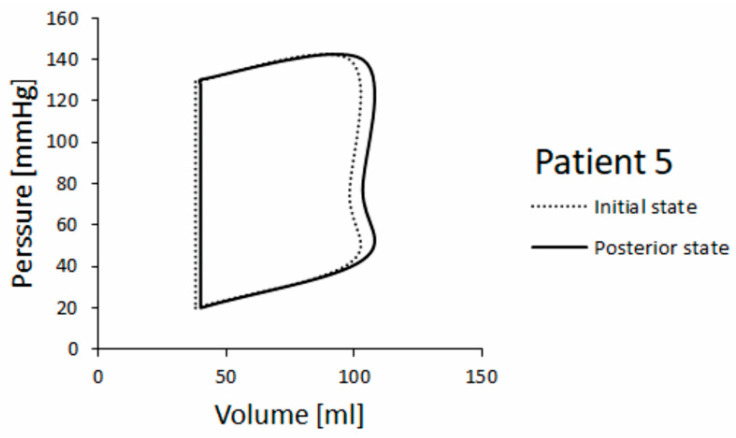
P-V diagram for patient 5.

In addition, P-V diagrams obtained for patients 3 and 5 are shown in Figure 12 and Figure 13, respectively. Pressure change is similar in both patients, but for patient 5 we found a larger volume change at posterior state (follow up) compared with patient 3. Patient 3’s state got worse, so the posterior state shows a decreased volume change between end-diastole and end-systole in P-V diagram.

Left ventricle ejection fractions (LVEFs) in the initial (baseline) and posterior (follow-up) state for HCM patients 3 and 5 are shown in Table 2 (clinical and simulation values). The LVEF was calculated as LVEF = (EDV − ESV)/EDV, where EDV is end-diastolic volume and ESV is end-systolic volume [45]. It can be seen that the simulated LVEF in the posterior state remains almost unchanged for patient 5, but it is lowered for patient 3 whose real health state was worse. Both findings are in accordance with clinical observation.

In clinical practice, the LVEF is useful for assessment of patient condition, but for a precise diagnosis cardiologists need additional medial information, knowing that the LVEF might be normal or even high but that this does not mean that much blood is being pumped out [46]. For that purpose, results and developed methods from in silico clinical trials can be applied, assisting in visualization of additional biomechanical parameters which cannot be measured in vivo, as well as in improved risk assessment and therapy directions for specific patients. 

### 3.2. Simulations of the Effect of Drugs on Improving State of HCM and DCM Heart Models (PAK FE Solver Coupled with MP Surrogate Model)

Simulations of the effect of drugs on improving the performance of HCM and DCM included the drugs that affect calcium transients (Disopyramide and Digoxin) and changes in kinetic parameters (Mavacamten and dATP). All simulations were performed using coupled PAK FSI, FE solver, and MP surrogate model. For this purpose, we created additional HCM and DCM parametric models of LVs using patient-specific measurements [16]. The boundary conditions were scaled and applied according to the patient-specific measures.

**Figure 14:**
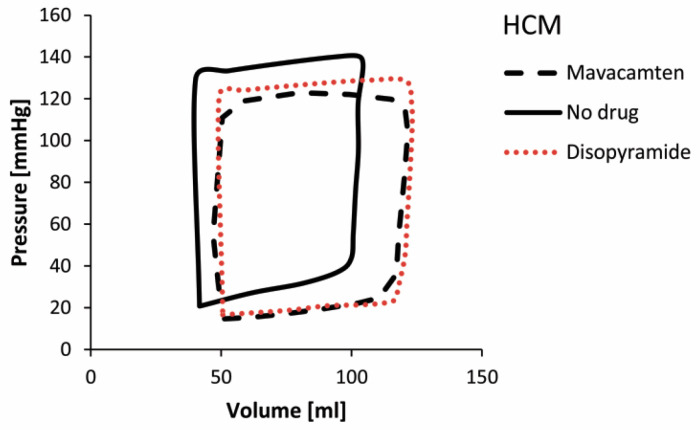
P-V diagrams for HCM at basic condition (without administered drug) and with using drugs Disopyramide and Mavacamten.

**Figure 15:**
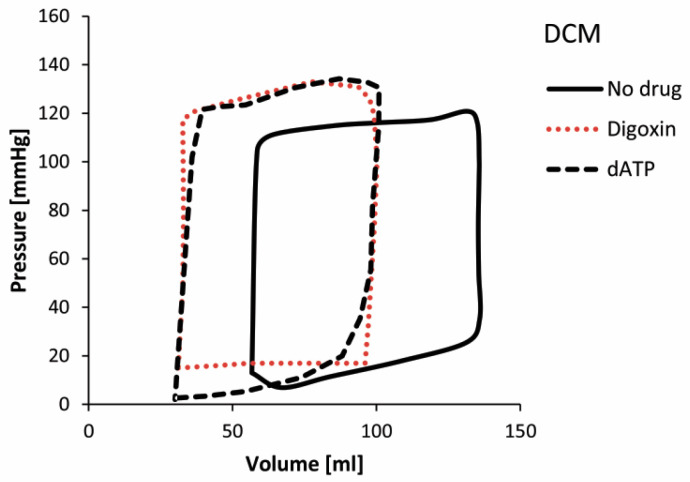
P-V diagrams for DCM at basic condition (without administered drug) and with using drugs Digoxin and dATP.

P-V diagrams for the HCM LV model at basic condition (without administered drug) and with using drugs Disopyramide and Mavacamten are presented in Figure 14, while P-V diagrams for the DCM LV model at basic condition and with administration of Digoxin and dATP are presented in Figure 15.

The predicted P-V diagram for HCM (Figure 14) at basic condition (without administered drug) shows lower volumes and higher ventricular pressures than normal, with reduced LVEF (LVEF = 59.33%) [47]. On the other hand, the simulation for DCM (Figure 15) at basic condition predicted lower ventricular pressure caused by increased size of the LV, thinner ventricle walls, and reduced contractility of DCM. Due to increased LV size, the P-V loop for the DCM model without administered drug is shifted toward lager ventricular volume, with LVEF = 56.83%.

The principal effects of drugs on HCM are a decrease in peak pressures and a shift of P-V loops toward higher volumes (Figure 14) and higher LVEFs. On the other hand, the effects of drugs on DCM (Figure 15) show an increase in ventricular peak pressures and LVEFs, while the P-V loops are shifted toward decreased volumes, corresponding to healthy hearts. Taken together, for the DCM we acquired larger volume change than for the HCM which was previously confirmed in clinical observations [47]. It can be observed that simulated drug effects shift P-V diagrams closer to basic conditions, which is a promising result for further investigations in optimization of drug therapy for specific cardiomyopathy patients.

**Figure 16:**
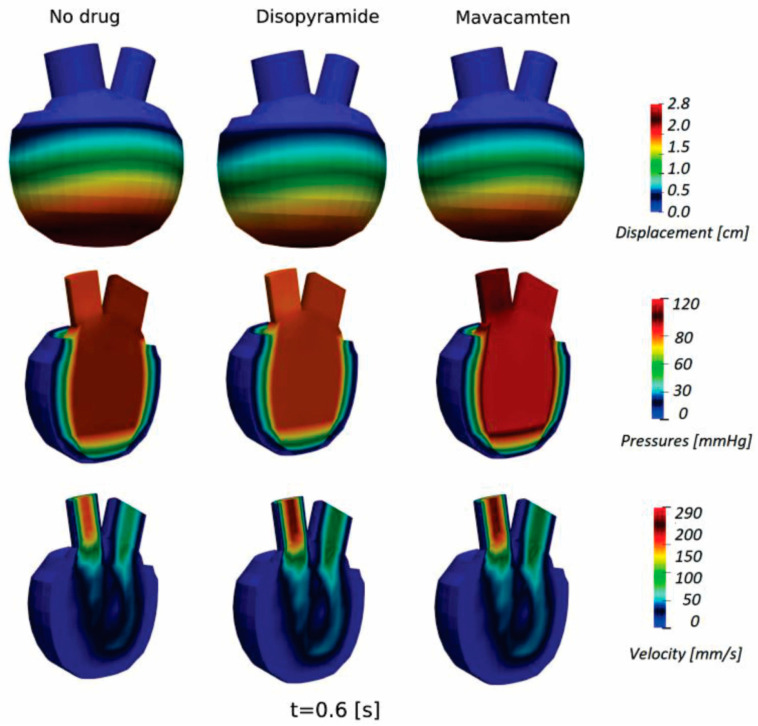
Displacement, pressures, and velocity distribution (peak systole, t = 0.6 s) for HCM at basic condition (without administered drug) and with using Disopyramide and Mavacamten.

**Figure 17:**
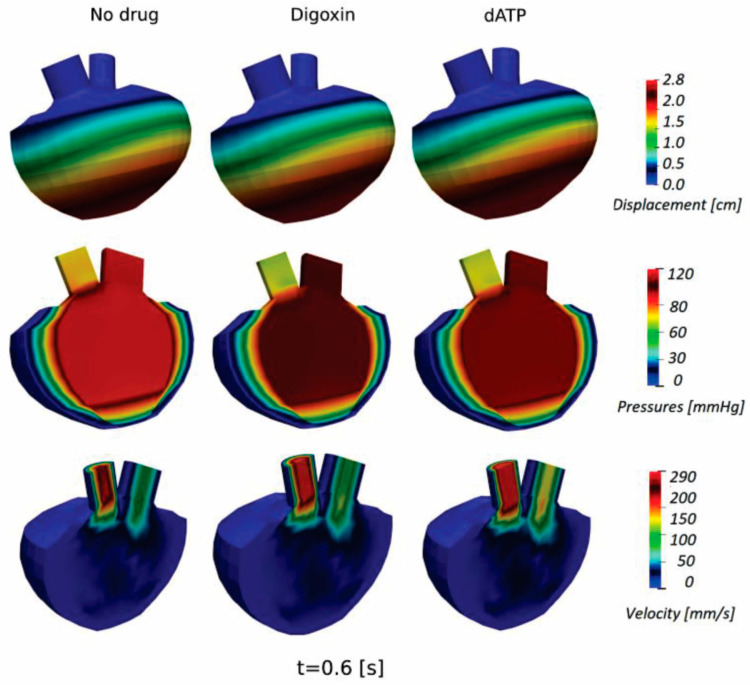
Displacement, pressures, and velocity distribution (peak systole, t = 0.6 s) for DCM at basic condition (without administered drug) and with using Digoxin and dATP.

In addition, displacements, pressures, and velocity distribution for the HCM LV model at basic condition (without administered drug) and with using Disopyramide and Mavacamten are presented in Figure 16. Displacements, pressures, and velocity distribution for the DCM LV model at basic condition (without administered drug) and with using Digoxin and dATP are presented in Figure 17. These parameters are shown at peak systolic moment (t = 0.6 s) for both the HCM and DCM LV models. The presented results provide additional insight into the changed distribution of biomechanical parameters without and with administered drugs on HCM and DCM LV models, while their visualization can assist in more detailed prognosis and directions of drug therapy for specific patients. 

## 4. Discussion and Conclusions

The SILICOFCM platform as a whole enables in silico animal and clinical trials for testing the effectiveness of pharmacological treatment for LV heart performance. Such an approach can significantly reduce the time and cost of running real animal and clinical trials for drug development and optimal testing which is one of main motivations for this study. The presented study is performed using coupled macro- and microsimulation through FE modeling of FSI and molecular drug interactions with the cardiac cells, as part of the SILICOFCM project [14]. The FSI algorithm within the PAK software was used for modeling the LV with nonlinear material model, together with stretches and integration along muscle fibers.

The study presents results of (i) SILICOFCM Risk Stratification Tool and PAK software for HCM patients at baseline and follow-up, as well as of (ii) simulated drug effects on the HCM LV model and the DCM LV model. The results closely follow the available clinical observations, which is a promising step for further improvement of computational methods also including a larger group of cardiomyopathy patients. 

In the case of drug modeling, two major groups are described by the principal action of specific drugs on modulating calcium transients and changing the kinetics of contractile proteins. According to the principal actions of drugs on the electro-mechanics LV coupling, simulations were separated in two scenarios. The effects of Disopyramide and Dygoxin, which modulate Ca^2+^ transients, were included in the first scenario, while Mavacamten and dATP, which affect changes of kinetic parameters, were included in the second scenario. Changes of pressures, displacements, and velocity distributions, as well as P-V loops in the LV models of HCM and DCM patients, are presented. The results provide a quantitative assessment of the effects of different drugs (Disopyramide and Dygoxin, Mavacamten and dATP) on cardiac output, including both systolic and diastolic LV pressures and volumes, as well as the LVEF.

It should be emphasized that the performed simulations are based on simplified LV geometries. In the case of detailed and patient-specific models, FE analyses are very time-consuming, especially when muscle micromodels are included. In contrary, our models are patient-specific in terms of dimensions of specific LV components but are geometrically simplified in order to avoid manual construction of the FE meshes for a large number of patients. Since models are not anatomically precise, results may slightly differ from the real state of the patient’s cardiac health. Additional limitations of the study are the lack of details regarding fully physical and biological properties of the specific patient’s heart. Despite those limitations, the presented methods can be used together to obtain better insight into the cardiac health and optimal drug therapy for specific patients.

In summary, this study was designed to propose that developed computational models can mimic, on a macroscopic level, the behavior of patients under different stages and types of cardiomyopathy disease. Moreover, this approach can give much more information for the risk prediction of cardiac disease in specific patients and better insight into the estimated effects of drug therapy, leading to improved patient monitoring and treatment.

## Figures and Tables

**Table 1:** Report from the Risk Stratification Tool for the selected five patients.

Patient No.	Prediction	Reliability	PredictedError	ConfidenceMin	ConfidenceMax
1	0.010995677	0.987413164	0.082859018	0.003019494	0.059243858
2	0.082876824	0.965970863	0.144172512	0.019990185	0.165266529
3	0.55732954	0.858801931	0.450618253	0.117242657	0.701754749
4	0.027759297	0.980057329	0.103892767	0.004188759	0.077506609
5	0.5642663	0.885699762	0.373704863	0.047019321	0.564266324

**Table 2:** LVEF of patients 3 and 5 in initial and posterior state compared with clinical observations.

Patient No.	LVEF [%]			
	Clinical Value − Initial	Simulation − Initial	Clinical Value − Posterior	Simulation − Posterior
3	64	59.75	50	49.10
5	60	61.42	60	61.4

## Data Availability

Data and materials for the cases running in this manuscript are deposited at www.silicofcm.eu (accessed on 6 February 2023) for consortium members. The data are available on request from the corresponding author.
